# Mastering Your fellowship: Part 4, 2023

**DOI:** 10.4102/safp.v65i1.5762

**Published:** 2023-06-01

**Authors:** Mergan Naidoo, Klaus von Pressentin, Tasleem Ras, Gert Marincowitz

**Affiliations:** 1 Department of Family Medicine, College of Health Sciences, University of KwaZulu-Natal, Durban, South Africa; 2 Department of Family, Community and Emergency Care, Faculty of Health Sciences, University of Cape Town, Cape Town, South Africa; 3 Department of Family Medicine, Faculty of Health Sciences, University of Limpopo, Polokwane, South Africa

**Keywords:** family physicians, FCFP (SA) examination, family medicine registrars, postgraduate training, national exit examination, emergency care

## Abstract

The ‘Mastering your Fellowship’ series provides examples of the question format encountered in the written and clinical examinations, Part A of the Fellowship of the College of Family Physicians of South Africa (FCFP [SA]) examination. The series is aimed at helping family medicine registrars prepare for this examination.

## Introduction

This section in the *South African Family Practice* journal is aimed at helping registrars prepare for Part A of the Fellowship of the College of Family Physicians of South Africa (FCFP [SA]) examination and will provide examples of the question formats encountered in the written examination: multiple choice question (MCQ) in the form of single best answer (SBA – Type A) and/or extended matching question (EMQ – Type R); short answer questions (SAQ), questions based on the critical reading of a journal (evidence-based medicine), and an example of an objectively structured clinical examination (OSCE) question. Each of these question types is presented based on the College of Family Physicians blueprint and the key learning outcomes of the FCFP programme. The MCQs are based on the 10 clinical domains of family medicine, and the SAQs will be aligned with the five national unit standards. The critical reading section will include evidence-based medicine and primary care research methods.

This edition is based on unit standard 1 (critically reviewing new evidence and applying the evidence in practise, leadership, and governance), unit standard 2 (evaluate and manage a patient according to the bio-psycho-social approach) and unit standard 5 (conduct all aspects of healthcare in an ethical, legal and professional manner). The domain covered in this edition is emergency care. We suggest you to attempt to answer the questions (by yourself or with peers or supervisors) before finding the model answers online: http://www.safpj.co.za/.

Please visit the Colleges of Medicine website for guidelines on the Fellowship examination: https://www.cmsa.co.za/view_exam.aspx?QualificationID=9.

We are keen to hear about how this series assists registrars and their supervisors in preparing for the FCFP (SA) examination. Please email us (naidoom@ukzn.ac.za) your feedback and suggestions.

## Multiple choice question (MCQ): Single best answer

A 65-year-old female known with type 2 diabetes mellitus, hypertension, dyslipidaemia, and chronic kidney disease presents to the emergency centre (EC) with respiratory distress. She has had multiple previous admissions for her medical problems with deteriorating kidney function. She was reviewed by the nephrology department, which found her unsuitable for the chronic renal programme. Today, she presents with metabolic acidosis (pH = 7.14), fluid overload, and a depressed level of consciousness. Her estimated glomerular filtration rate is 6 mL/min per 1.73 m^2,^ which is her normal baseline. You counsel the family. What is the most appropriate next step?

- Admit her to the general ward and treat her symptomatically.
- Re-discuss her case with the nephrology department.
- Refer her to the intensive care unit at the regional hospital.
- Refer her to the regional hospital for compassionate dialysis.
- Start furosemide and bicarbonate infusions and admit her to the high-care unit.

*Answer: a*)

Unfortunately, scenarios like the one sketched here are too common in many healthcare settings. The massive increase in non-communicable diseases (NCDs) with the impact on personal health and the consequent burden on the healthcare system has resulted in many ECs being inundated with patients presenting at the end of life. For many clinicians who intuitively want to intervene and help the patient, the response is often to subject these patients to medical and other interventions to prolong life. Dealing with a dying patient is emotionally taxing for many emergency care practitioners, so ensuring that the institution has good palliative care guidelines, which include psychosocial support for staff, is critical. Ideally, discussions with such patients should have started when the chronic disease was diagnosed, and the palliative care interventions needed to have escalated as the effects of the disease process progressed. The multidisciplinary team should have discussed issues such as patient’s and family’s fears and concerns, advance directives, the patient’s views on resuscitation and dealing with unfinished business much earlier. The patient’s choice of whether they wanted resuscitation or not must be documented in the clinical notes. Involvement of spiritual support, if appropriate, is also a key consideration.

In situations where treatment is considered futile, and the prognosis is poor, healthcare workers have no legal duty to provide treatment. The World Medical Association Medical Ethics Manual provides some guidance for doctors by stating that:

[*A*] doctor has no obligation to offer a patient futile or non-beneficial treatment’ and describes treatment as ‘medically futile’ when it’ offers no reasonable hope of recovery or improvement or because the patient is permanently unable to experience any benefit.

The decision not to provide futile treatment results in conflicts in our ethical principles of autonomy, beneficence, non-maleficence, and justice. It is essential that the patient’s family be counselled on the harms of prolonging life and extending suffering. Another important consideration is the equitable distribution of scarce medical resources, which may compromise access to others dependent on such care. This relates to the principle of justice.

The given scenario is a case of medical futility, so extending life will prolong suffering for both the patient and the family. It is essential to discuss the situation with the family so that they are fully aware of the clinical decision-making. Notably, one should sensitively address their fears and concerns. Implementing palliative care practice guidelines at healthcare institutions allows one to deal with death and dying proactively, and prepares the patient and family for the inevitable.

**Further reading**

- Gwyther L. Chapter 4.6.6: Palliative care. In: Mash RJ, editor. Handbook of family medicine. 4th ed. Cape Town: Oxford University Press, 2017; p. 129–131.
- McQuoid-Mason DJ. Should doctors provide futile medical treatment if patients or their proxies are prepared to pay for it? SAMJ: S Afr Med J. 2017;107(2):108–109. https://doi.org/10.7196/SAMJ.2017.v107i2.12191

## Short answer question (SAQ)

The family physician’s role as a leader of clinical governance in the domain of emergencies and trauma.

You recently started working as a family physician at a district hospital. You are requested to do a record audit on two patients who died recently in casualty. You notice that both patients were trauma patients (the first patient sustained a stabbed chest and the second patient sustained bilateral femur fractures following a motor vehicle accident). They waited more than an hour before the doctor saw them. You were told that the casualty was very busy on both occasions. The clinical manager now asks you to address the problem:

1. List six key pieces of information you would look for in the patient files when doing the record audit on the two patients who died in casualty. (6 marks)
2. As part of the quality improvement process, you and the team want to investigate all possible reasons for the significant delay in attending to seriously injured patients. You decide to use ‘Process Mapping’ for this purpose. Mention at least six different points a patient must pass through in an average district hospital casualty that you would like to assess during your process mapping. (1/2 mark for each suggested point of contact in the casualty process) (3 marks)
3. As discussed here, mention two aspects that you and your team want to assess at each point the patient must pass through. (2 marks)
4. Name three other things that could go wrong during this process apart from the time delay. (3 marks)
5. From the ‘process mapping’, you identify a significant delay at several points that patients had to go through before the doctor saw them. Describe how you will go about planning an intervention. (2 marks)
6. Suggest two possible interventions to improve the delay in care in casualty. (2 marks)
7. Explain how you will implement the plan in the casualty department of your hospital. (2 marks)

Total: 20 marks

Model answers

**1. List six key pieces of information you would look for in the patient’s files while performing the record audit on the two patients who died in casualty. (6 marks)**

(*Check and document any of the following from the patient files to understand the possible cause of the death and contributing factors. Give one mark for any of the 6 points listed below*)

Look specifically for factors that influenced the time from the accident to when the patient was seen by the doctor (resuscitation). Both patients are classified as having severe injuries that have to be seen immediately (a severe chest injury and other bilateral femur fractures):

- Mode of transport: Arrival by ambulance or brought by the family. (1 mark)
- Timeline: Between accident and arrival at hospital EC, to first evaluation and/or triage; waiting time before being seen by the doctor; waiting time before resuscitation. (1 mark)
- Triage at arrival: What observations and/or examination was performed at the triage point? (1 mark)
- What was the response to the patient’s condition at triage, and was it according to resuscitation guidelines: severe trauma patients are classified as Priority 1 and should be seen immediately. (1 mark)
- Review the clinical notes of the initial assessment by the doctor of these patients with severe trauma (chest trauma and bilateral fractured femurs). Evaluate the history, examination, assessment, and management that was recorded in the file with specific reference to airway, breathing, and circulation assessment. Specifically evaluate if there is any assessment if the patient had any respiratory distress or was any of them in shock, for example, respiration rate, pulse rate, blood pressure, and oxygen (O_2_) saturation. (1 mark)
- Review the notes on all the resuscitation efforts and evaluate if it was according to acceptable protocols (Advanced Trauma Life Support [ATLS] guidelines). Review the details of resuscitation: From the patient’s file try to identify how the resuscitation was carried out with specific reference to the airway, breathing and circulation (ABC) guidelines. Assess what was performed to assist the airway and breathing (especially for the patient with a stabbed chest) and check if two intravenous (IV) lines were inserted. Evaluate how the continuous monitoring was carried out. (1 mark)

Attempt to understand the possible cause of death for both patients:

- Evaluate the patient’s file and try to establish the clinical cause of death (1 mark).
- Look for the post-mortem report and document the cause of death as given by the post-mortem findings (1 mark).

Any other appropriate information not listed here may also be considered for marks.

**2. As part of the quality improvement proc ess, you and the team want to investigate all possible reasons for the significant delay in attending to seriously injured patients. You decide to use “Process Mapping” for this purpose. Mention at least 6 different points a patient must pass through in an average district hospital casualty that you would like to assess during your process mapping. (3 marks)**

*(1/2 mark for each suggested point of contact in the casualty process)*

- Arrival at the hospital by ambulance or private transport.
- Next step: administration for file.
- Next step: observation room or emergency room.
- Next step: seen by a doctor.
- Next step: special investigations: X-ray or blood tests.
- Next step: management, getting treatment.
- Next step: transfer to a ward or to the pharmacy (discharges); waiting period at the pharmacy before receiving discharge medications/transported to the ward.
- Next step: arrival in the ward

**3. As mentioned here, mention two aspects you and your team want to assess at each point the patient must pass through. (2 marks)**

- Evaluate the average time spent per step. (1 mark)
- Identify what happens at each step, what decisions are made at each step, does the step add value for the patient. (1 mark)

**4. Name three other things that could go wrong during this process apart from the time delay. (3 marks)**

(*The first 2 points mentioned here are compulsory to mention for 2 marks. For the 3rd mark any of the remaining points can be mentioned*)

- *Patient’s condition deteriorates unnoticed during the waiting period.*
- *Inability to prioritise severely ill/injured patients – P1.*
- Not assisted and just waiting.
- Load shedding, off-line computers: what is supposed to happen at this point cannot be done.
- Asked to wait for blood results, X-rays.
- The patient gets forgotten or lost in between different places and/or points.
- Patient treated in a non-patient-centred manner.

**5. From the ‘process mapping’ you identify a significant delay at several points patients had to go through before the doctor saw them. Describe how you will go about planning an intervention. (2 marks)**

(*Mention any of the points below for 2 marks*)

- Present the data from the process mapping to your team (1 mark)
- Compare your data (findings from the process mapping) with expectable international standards: for example, compare the waiting time for P1, P2 and P3 patients with the waiting time as set by the standards for example, ALTS guidelines. (1 mark)
- Identify what to change: Identify P1 patients as patients who cannot wait in the queue and need resuscitation immediately. (1 mark)

**6. Suggest two possible interventions to improve the delay in care in casualty. (Mention any of the points below for 2 marks)**

- Implement a Triage system in casualty.
- Develop guidelines for the management of severely injured patients.
- Display resuscitation guidelines and protocols on a notice board in casualty.
- Do in-service training on assessment, resuscitation, and management of severely injured patient.

**7. Explain how you will implement the plan in the casualty department of your hospital. (Mention any of the points below for 2 marks)**

- Get the cooperation and buy-in from the team at large including managers (casualty, nursing manager, clinical manager) and all the staff members involved.
- Train all the staff categories: clerks, nurses and doctors on the intervention, for example, interpreting observations, classifying patient priorities, and acceptable time (triage system).
- Develop a monitoring/audit system to ensure changes have been implemented.

**Further reading**

- Merrick C (executive editor). ATLS. 10th ed. Chicago, IL: American College of Surgeons; 2018, p. 2–81 & 148–167.
- Mazaza S, Gurst C. Chapter 8.9 & 8.10. Leadership and clinical Governance. In: Mash B, editor. Handbook of family medicine. 4th ed. Cape Town: Oxford University Press; 2017, p. 372–382.

## Critical appraisal of research

Read the accompanying article carefully and then answer the following questions (*total 30 marks*). As far as possible, use your own words. Do not copy out chunks from the article. Be guided by the allocation of marks concerning the length of your responses.

- Straeuli C, Jenkins L, Droomer N. Patients requiring palliative care attending a regional hospital emergency centre in South Africa: A descriptive study. Afr J Emerg Med. 2022;12(4):387–392. https://doi.org/10.1016%2Fj.afjem.2022.08.006

1. Critically appraise the authors’ choice of study design to answer the research question and limit bias. (6 marks)
2. Critically appraise the description of the study population. (3 marks)
3. Critically appraise the description of the sample size calculation. (3 marks)
4. Critically appraise the description of the sampling strategy. (4 marks)
5. Critically appraise the measurement of the outcomes of interest. (3 marks)
6. Comment on whether the study period selection may have introduced potential confounding factors. (3 marks)
7. Critically appraise the appropriateness of the statistical analysis. (2 marks)
8. Use a structured approach (e.g. READER) to discuss the value of these findings to your practice. (6 marks)

Model answers

**1. Critically appraise the authors’ choice of study design to answer the research question and limit bias. (6 marks)**

- The authors described the study design as a single-centre prospective, descriptive study. They wished to describe the prevalence of the outcome variable of interest: the sub-population of patients who would benefit from palliative care, who form part of the so-called ‘caseload’ or patients presenting to an emergency centre in a large regional hospital in the Western Cape.
- The authors argued that their choice of study design was based on the fact that they were familiar with the study setting and wanted to limit ‘bias by doing a prospective (as opposed to a retrospective) study’.
- When considering the strength and appropriateness of this study design, one may conclude that this descriptive, cross-sectional design is appropriate as it can assess the prevalence of an outcome of interest such as estimating the prevalence of patients with potential palliative care needs attending the emergency centre.
- However, the authors’ choice of a prospective design to ‘limit bias’ is not clearly described, as the type of bias was not specified. Furthermore, a descriptive study design does not allow for causal inference or generalisation beyond the specific population studied.
- An example of a prospective observational study is typically a cohort study in which selection bias may occur when deciding on the method of measuring exposure to select exposed and non-exposed individuals. In a retrospective observational study, information bias may occur when existing records are used, which may be complicated by missing information.
- One may assume that the authors decided on a prospective design to ensure that information bias may not be an issue as they wished to ensure that the emergency centre clinicians have been sensitised to the study, and that potential patients in need of palliative care are correctly identified and coded in the electronic health records.

**2. Critically appraise the description of the study population. (3 marks)**

- It is important to define the study population before considering how to sample from it. It is also helpful to define a target population (the broadest population to generalise findings) and the study population (the population accessible to the researchers). The study population should be described in terms of people, place and time.
- In this article, the researchers described the study population as all patients entering the emergency centre at this regional hospital during 3 months (November 2020 – January 2021). The population description of this study is therefore well-described.
- The researchers also provided detailed information on the study setting from which the target population is drawn, namely the practice makeup of the emergency centre in terms of staffing, the range of clinical disciplines represented, as well as the mix of presentations (from referred patients from surrounding district hospitals to presentations from the local population of the sub-district, which relies on George regional hospital for its services, including after-hours).

**3. Critically appraise the description of the sample size calculation. (3 marks)**

- The authors described a sample size calculation used in consultation with a biostatistician, which showed that a minimum sample of 270 cases would be required to detect a difference in proportions of more than 30% between two sub-groups of equal size. Sample sizes may be calculated for either a difference in means or a difference in proportions between equal-sized groups.
- Insufficient information is provided on the two sub-groups, which were considered (presumably those patients presenting to the emergency centre with palliative care needs and those without palliative care needs).
- It is also unclear why the value 30% was selected as the difference in proportions. The difference in proportions may have been based on the difference in scores between these groups based on the shortened Palliative Care Identification tool (or the predicted mortality based on the tool’s initial screening question, ‘would you be surprised if the patient were to die in the next year?’). It would also be useful to provide a reference to a study, which supports this decision – presumably, this decision was based on the study that validated the tool for South Africa (reference 13 in the article).

**4. Critically appraise the description of the sampling strategy. (4 marks)**

- As it is seldom possible to collect data from the entire study population, a representative sample must be selected from which data will be collected. Unfortunately, the sampling strategy in this study is not very clear to the reader. However, the inclusion and exclusion criteria appear to be clearly defined.
- It appears that the authors employed a two-step sampling approach, namely an inclusion assessment by the treating clinician using a validated instrument (the shortened Palliative Care Identification tool) to ensure that the patients in need of palliative care are identified and captured on an online electronic information system used in the emergency centre (HECTIS), followed by a review of the patients’ electronic patient records captured on a different system (ECM), to validate that these patients met the criteria of the validated instrument.
- Inclusion criteria were applied during the initial assessment: all patients presenting to the emergency centre during the study timeframe were assessed for inclusion: patients were included if they were ‘over the age of 18 years’ (presumably 18 years and older) and met the criteria of the screening tool.
- Exclusion criteria were applied during the second step when the electronic records were reviewed to confirm the correct coding during the clinician’s initial assessment. Patients not meeting the shortened tool criteria: aged younger than 18 years or had insufficient records were excluded.

**5. Critically appraise the measurement of the outcomes of interest. (3 marks)**

- The key outcome of interest is to identify patients with potential palliative care needs attending the emergency centre. The methods section describes the steps the research team took to measure this outcome consistently during the study period.
- A tool developed and validated for low- and middle-income countries such as South Africa (with its high burden of HIV and tuberculosis) was used. This tool was based on a Gold Standards Framework Prognostic Indicator Guidance identification tool, which aids clinicians to identify patients who have a high risk of death in the next 12 months and a potential palliative care need. This tool has a simple checklist format, which the authors describe as being easy to use by non-palliative care clinicians such as those working in the study setting. Clinicians working in the study setting received training from a doctor experienced in palliative care to ensure that patients were correctly coded based on the shortened screening tool. During the electronic records review, the inclusion of patients with palliative care needs was validated based on variables extracted from the records.
- The research team also performed an internal validity assessment during the first month of data collection by randomly selecting and reviewing 100 patient files, which helped them to assume a 90% accuracy in identifying patients with the outcome of interest.

**6. Comment on whether the study period selection may have introduced potential confounding factors. (3 marks)**

- The study period selection may have introduced potential confounding factors, some of which are mentioned by the authors in the limitations section. The study was conducted over 3 months, during which South Africa experienced a wave of severe acute respiratory syndrome coronavirus 2 (SARS-CoV-2) infections. The authors stated that the diagnosis of SARS-CoV-2 was not included as a criterion for identifying patients with potential palliative care needs in order to maintain external validity.
- However, the pandemic and associated restrictions may have affected patient’s attendance at the emergency centre and their need for palliative care. For example, patients may have hesitated to seek medical care because of fear of exposure to coronavirus disease 2019 (COVID-19) or experienced challenges with accessing healthcare services when healthcare resources may have been diverted towards managing COVID-19 cases.
- Furthermore, a 3-month period is too short to compensate for seasonal variation, especially as this was during the summer months (the reasons for presenting in the winter months may be very different). In addition, the change-over of emergency centre staff may also have introduced selection bias as a new group of intern and community service doctors started during the study period.

**7. Critically appraise the appropriateness of the statistical analysis. (2 marks)**

- The statistical analysis used in this study appears to be appropriate for the research question and data collected.
- The authors followed general analysis guidelines for descriptive analyses, comparing continuous and categorical variables, used appropriate software for analysing the data, and provided clear descriptions of their data analysis methods.

**8. Use a structured approach (e.g., READER) to discuss the value of these findings to your practice. (6 marks)**

The READER format may be used to answer the following questions:

- Relevance to family medicine and primary care?
- Education – does it challenge existing knowledge or thinking?
- Applicability – are the results applicable to my practice?
- Discrimination – is the study scientifically valid enough?
- Evaluation – given the above, how would I score or evaluate the usefulness of this study to my practice?
- Reaction – what will I do with the study findings?

*The answer may be a subjective response but should be one that demonstrates a reflection on the possible changes in the student’s practice within the South African public healthcare system. It is acceptable for the student to suggest how their practice might change, within other scenarios after graduation* (*e.g. privat e general practice). The reflection on whether all important outcomes were considered is, therefore, dependent on the reader’s perspective (is there other information you would have liked to see*?).

*A model answer could be written from the perspective of the family physician employed in the South African district health system:*

- R: This study is relevant to the African primary care context because many patients with unmet and undiagnosed palliative care needs often present in emergency centres at district and regional hospitals.
- E: This study highlighted the shortened screening tool’s usefulness in identifying patients with possible palliative care needs. It also illustrates that screening should be complemented with the training of emergency centre staff and strengthening community-based palliative care services.
- A: It is not possible to generalise the study’s findings to the broader South African setting, given the challenges with confounding factors and internal study limitations.
- D: Regarding discrimination, the study has limitations because of the effect of the COVID-19 pandemic, the short study period, which will not account for seasonal variation, and the emergency centre staff change-over during the study period.
- E: The study described the prevalence of patients with potential palliative care needs in the emergency centre and common reasons for attendance. The study’s limitations hamper its usefulness but could serve to sensitise emergency centre staff and management to the need for screening using the shortened tool.
- R: Although the study has limitations, the challenge of identifying patients presenting to emergency centres with their unmet palliative care needs could warrant a review of current health service access to palliative care. These preliminary findings may be helpful for follow-up research with a more robust design as well as service-strengthening and advocacy activities.

Total: 30 marks

**Further reading**

- The Critical Appraisals Skills Programme (CASP). CASP checklists [homepage on the Internet]. 2023 [cited 2023 Mar 23]. Available from: https://casp-uk.net/casp-tools-checklists/
- Joanna Briggs Institute (JBI). Critical appraisal tools [homepage on the Internet]. 2023 [cited 2023 March 23]. Available from: https://jbi.global/critical-appraisal-tools
- Pather M. Evidence-based family medicine. In: Mash B, editor. Handbook of family medicine. 4th ed. Cape Town: Oxford University Press; 2017, p. 430–453.
- Schuster T. Chapter 20. How to conduct observational studies. In: Goodyear-Smith F, Mash B, editors. How to do primary care research. Boca Raton, FL: CRC Press; 2019, p. 195–202.
- Mash B, Ogunbanjo GA. African primary care research: Quantitative analysis and presentation of results. Afr J Prim Health Care Fam Med. 2014;6(1):1–5. https://doi.org/10.4102/phcfm.v6i1.646
- Govender I, Mabuza LH, Ogunbanjo GA, Mash B. African primary care research: Performing surveys using questionnaires. Afr J Prim Health Care Fam Med. 2014;6(1):1–7. https://doi.org/10.4102/phcfm.v6i1.589
- MacAuley D. READER: an acronym to aid critical reading by general practitioners. Br J Gen Pract. 1994;44(379):83–85.

## Objectively structured clinical examination (OSCE) station: Emergencies

### The objective of the station

This station tests the candidate’s ability to supervise a junior colleague in managing a patient with a hyperglycaemia emergency.

## Requirements

Simulated patient: adult male/female.

## Instructions for candidate

You are the family physician working at a district hospital. On the handover ward round, the following patient is discussed.

## Your task

- Please respond to your junior colleague’s request for assistance.
- You do not need to examine this patient. All examination findings will be provided on request.

## Instructions for the examiner

**FIGURE 1:**
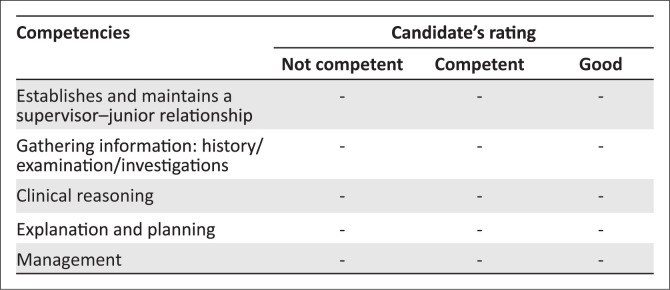
Marking template.

- This is an integrated consultation station in which the candidate has 15 min. Familiarise yourself with the assessor guidelines detailing the expected responses from the candidate.
- No marks are allocated. In the mark sheet (Figure 1), tick off one of the three responses for each competency listed. Ensure that you are clear on the criteria for judging a candidate’s competence in each area.

## Guidance for examiners

The aim is to establish that the candidate has an effective and safe approach to managing hyperglycaemic emergencies in an adult.

*Not competent*: Patient safety is compromised (including ethically and legally), or the task is not completed.

*Competent*: The task is completed safely and effectively.

*Good*: In addition to displaying competence, the task is completed efficiently and in an empathic, patient-centred manner (acknowledges patient’s ideas, beliefs, expectations, concerns/fears).

1. **Establishes and maintains a good collegial relationship**

The competent candidate is respectful and engages with the colleague dignifiedly.

The good candidate is empathic, compassionate and collaborative, facilitating active participation in key areas of the consultation.

2. **Gathering information**

The competent candidate gathers sufficient information to establish a working diagnosis (hyperosmolar hyperglycaemic state – HHS).

The good candidate additionally has a structured and holistic approach (explores baseline knowledge and understanding of the colleague; explores ongoing risk factors; uses evidence-based bedside teaching approach like 1-min preceptor or similar).

3. **Clinical reasoning**

The competent candidate identifies the diagnosis (HHS) and acknowledges the difficulty in making a diagnosis for a junior clinician.

The good candidate makes a specific diagnosis (HHS) and has a structured approach to addressing the colleague’s development (acknowledges clinical complexity; empathises with how overwhelming the emergency centre can be; identifies gaps in teaching).

4. **Explaini ng and planning**

The competent candidate uses clear language to explain to the colleague and uses strategies to ensure the colleague’s understanding (questions OR feedback OR reverse summarising).

The good candidate additionally ensures that the colleague is actively involved in decision-making, paying particular attention to knowledge-sharing and empowerment, given the emergency of the situation and the further assessments needed to confirm the diagnosis.

5. **Management**

The competent candidate proposes appropriate intervention (in-patient fluid management given dehydration and hypernatremia; attention to potassium supplementation; insulin infusion).

The good candidate discusses treatment risks, frequently made mistakes, and facilitates a structured follow-up plan. Learning resources and mentoring opportunities in future are identified to assist the colleague with ongoing development.

## Role play: Instructions for actor

Adult male/female: medical intern

**Opening statement**

‘Doctor, I saw this 64 years old patient, a known diabetic, who now has diabetic ketoacidosis, but there are some puzzling things. She was admitted about 40 min ago. Can you please advise … ’.

**History**

**Open responses:** Freely tell the doctor …

- The patient is known with type 2 diabetes since age 50 years – on maximum oral agents, according to the folder (metformin 1000 mg twice daily and glibenclamide 5 mg daily).
- The family brought her in because she was becoming confused since yesterday.
- On admission, the sugar levels were high and unrecordable on fingerprick testing. Laboratory testing indicates 45.6 mmol/L.
- What puzzles you:
  ■ Blood ketones were negative, but urine ketones were positive.
  ■ Sodium was 156 mmol/L (normal ≤ 140 mmol/L).

**Closed responses**: Only tell the doctor if they brings this up:

- You started normal saline drip at 1000 mL/h – patient weighs about 70 kg.
- Not sure what to do about high sodium levels.
- Must you also start an insulin infusion? How to do this?
- You’ve assessed for urinary, skin or respiratory infections – none found.
- Full blood count was normal.

The family has left and no further history is forthcoming. The patient is too confused to give any more information.

**Ideas, concerns and expectations:** You’re very worried that there is something that you are missing.

**Further reading**

- Department of Health, South Africa. Standard treatment guidelines hospital level. Adult. 2019:8.14–8.16.

